# Underground salt and potash workers exposed to nitrogen oxides and diesel exhaust: assessment of specific effect biomarkers

**DOI:** 10.1007/s00420-022-01876-2

**Published:** 2022-05-18

**Authors:** Lisa Gamrad-Streubel, Lisa-Marie Haase, Katharina K. Rudolph, Katrin Rühle, Annette M. Bachand, Lori Crawford, Kenneth A. Mundt, Jürgen Bünger, Dirk Pallapies, Dirk Taeger, Swaantje Casjens, Anja Molkenthin, Savo Neumann, Jörg Giesen, Volker Neumann, Thomas Brüning, Thomas Birk

**Affiliations:** 1 Environment and Health, Ramboll Deutschland GmbH, City Tower-Limbecker Platz1, 45127 Essen, Germany; 2 Ramboll US Consulting, Inc, Amherst, MA USA; 3 Cardno ChemRisk, Boston, MA USA; 4 Institute for Prevention and Occupational Medicine of the German Social Accident Insurance, Institute of the Ruhr University Bochum (IPA), Bochum, Germany; 5 Institute for the Research On Hazardous Substances (IGF), Bochum, Germany

**Keywords:** Epidemiological study, Cross-sectional study, Salt and Potash mining, Diesel exhaust, Nitrogen oxides, Occupational exposure Limit

## Abstract

**Purpose:**

Occupational exposure limits (OEL) for nitrogen oxides (NO, NO_2_) and diesel exhaust (EC-DPM) were reassessed by the German authorities in 2016/2017. We performed a clinical cross-sectional study among salt and potash underground workers exposed to these substances at relatively high levels to examine possible indicators of acute effects on workers’ health.

**Methods:**

We measured post- versus pre-shift differences in cardiovascular, inflammatory, immune, and respiratory effect biomarkers and assessed their associations with personal exposures measured during the same shift. We also compared post- versus pre-shift differences in biomarker levels between exposure groups defined based on work site and job type.

**Results:**

None of the above-ground workers exceeded the OEL for NO_2_ and only 5% exceeded the OEL for EC-DPM exposure. Among underground workers, 33% of miners and 7% underground maintenance workers exceeded the OEL for NO_2_; the OEL for EC-DPM was exceeded by 56% of miners and 17% of maintenance workers.

Some effect biomarkers (thrombocytes, neutrophils, MPO, TNF-α, IgE, FeNO) showed statistically significant differences between pre- versus post-shift measurements; however, there were no consistent associations between pre- and post-shift differences and exposure group or personal exposure measurements during the shift.

**Conclusions:**

We did not find evidence of associations between workplace exposure to NO, NO_2_ or EC-DPM and clinically relevant indicators of acute cardiovascular, inflammatory and immune, or respiratory effects among salt and potash underground workers in Germany.

**Supplementary Information:**

The online version contains supplementary material available at 10.1007/s00420-022-01876-2.

## Introduction

Salt and potash miners are commonly exposed to blasting fumes and exhausts from machines and vehicles required for underground mining operations. Several previous studies showed that workers in underground potash mines in Germany had an increased incidence of respiratory symptoms, including reduced lung function (Lotz et al. 1998, 2006, 2008). These studies, begun in the mid-1990s, initially examined the correlation between respiratory disorders and exposure to salt dust. Later, when discussions began in Europe on new limits for air pollutants such as nitrogen monoxide (NO) and nitrogen dioxide (NO_2_), the research expanded to evaluate potential dose–response relationships between exposure to nitrogen oxides (NO_*X*_) and lung function. In a longitudinal study, Lotz et al. (2008) reported a dose–response relationship between salt mine exposures and decreased lung function. However, because the workers’ personal exposures to nitrogen oxides, salt dust, and diesel exhaust were mixed and highly correlated (with correlation coefficients as high as 0.99 for diesel exhaust and inhalable salt dust), it was not possible to identify the potential effects of any individual pollutants.

As recommended by the Scientific Committee on Occupational Exposure Limits (SCOEL) of the European Commission in June 2014, Occupational Exposure Limits (OELs) for NO_X_ were significantly reduced to 0.5 ppm for NO_2_ and 2.0 ppm for NO by the regulatory authorities in Germany in 2016 (Ausschuss für Gefahrstoffe 2021; Scientific Committee on Occupational Exposure Limits 2014a, b). For diesel particulate matter measured as elemental carbon (EC-DPM) a new OEL was set at 0.05 mg/m^3^ in 2017 (Ausschuss für Gefahrstoffe 2021). Underground salt and potash mines cannot easily comply with the new OELs in the short term (Kübler et al. 2016). However, one German mining company developed a 5-year transitional action plan to reduce exposures by implementing several technical measures such as improving ventilation and switching to electric vehicles. The present study represents a key part of this action plan and was conducted in collaboration with the mining company’s statutory accident insurance institution for trade and industry and a panel of occupational health scientists, epidemiologists, and biostatisticians. To investigate if the delay in exposure reduction would negatively affect underground workers’ health, we assessed whether exposures to current levels of NO, NO_2_ and diesel exhaust are associated with changes in indicators of acute health effects. In sensitivity analyses, we considered long-term pulmonary effects. We present findings based on two sites of the mining company.

## Material and methods

### Study participants

Based on power and sample size calculations from an initial feasibility study, we aimed to enroll approximately 1000 underground workers and 250 above-ground workers. Participants were eligible for inclusion if they had worked for at least one year at one of the two German sites of the above-mentioned salt and potash mining company. We excluded underground workers who had worked in a different mine for more than one year and above-ground workers who had previously worked underground for more than one year. Workers who suffered from occupationally unrelated chronic diseases such as ulcerative colitis were also excluded. Workers with acute hay fever symptoms on the day of examination were asked to participate at a later date. Due to insufficient numbers to present gender-specific results, 26 female workers were excluded from the study.

Exposures tended to be higher among underground workers at one site (Dahmann et al. 2007), likely due to differences in dust handling and consistency of the salt at the two plants. However, because the technology at both plants was comparable and the chemical composition of the salt dust was consistent (Lotz and Kersten 2012), we combined participants from both sites in the analyses. In sensitivity analyses, no differences in effect estimates between the sites could be detected.

### A-priori selection of exposure groups

Above-ground facility workers who experienced little exposure to NO_X_ or diesel exhaust served as the reference group. This means that those workers who are regularly occupationally exposed to higher levels of specific exposures such as diesel exhaust (e.g., through the operation of diesel-powered machines) were excluded from the study not to bias this group as reference. We subdivided underground workers into maintenance workers and miners. Due to differences in ventilation systems and in the amounts of diesel exhaust and blasting fumes in different areas of the mine, exposures among underground workers depended on workplace and activity, and miners were generally subject to higher exposure levels than underground maintenance workers. Neither underground nor above-ground workers were required to wear respiratory protection during their daily work.

### A-priori selection of biomarkers

Based on a broad literature review of potential biomedical endpoints, we focused on acute biomarkers of early cardiovascular disease (partial thromboplastin time (PTT), thrombocytes, blood pressure); acute biomarkers of inflammation and immunological responses (neutrophils, Interleukin 6 and 8 (IL-6 and IL-8), tumor necrosis factor α (TNF-α), myeloperoxidase (MPO), Club cell protein (CC16), C-reactive protein (CRP), Immunoglobulin E (IgE)); and one acute biomarker of lung and respiratory diseases (FeNO). Neutrophils and thrombocytes were analyzed in EDTA-treated blood, PTT was analyzed in citrate-treated plasma, and the remaining biomarkers were analyzed in serum.

We identified a reference range or cut-off point for normal values for each biomarker except for CC16, which is an experimental parameter with no reference range available. For biomarkers measured in blood or urine, reference ranges reflected 95% of the general population and were based on information from the manufacturer of the analytical assays and the analytical laboratory. The cut-off point for FeNO was based on existing literature (Dweik et al. 2011).

In sensitivity analyses, we assessed a biomarker of chronic lung and respiratory diseases. Specifically, we collected pre-shift measurements of FEV_1_% and FVC%, and we calculated FEV_1_%/FVC%, the percent predicted ratio of the Tiffeneau Index. The Tiffeneau Index (FEV_1_/FVC) is the ratio of forced expiratory volume in the first second (FEV_1_) to forced vital capacity (FVC) (Bhatt et al. 2019). The cut-off point for FEV_1_%/FVC%, standardized using reference values for FEV_1_ and FVC, is based on the GLI-2012 reference equations published by the ‘Global Lung Function Initiative’ (Cooper et al. 2017; Quanjer et al. 2012; Richter et al. 2008).

### Data collection

**Fig. 1:**
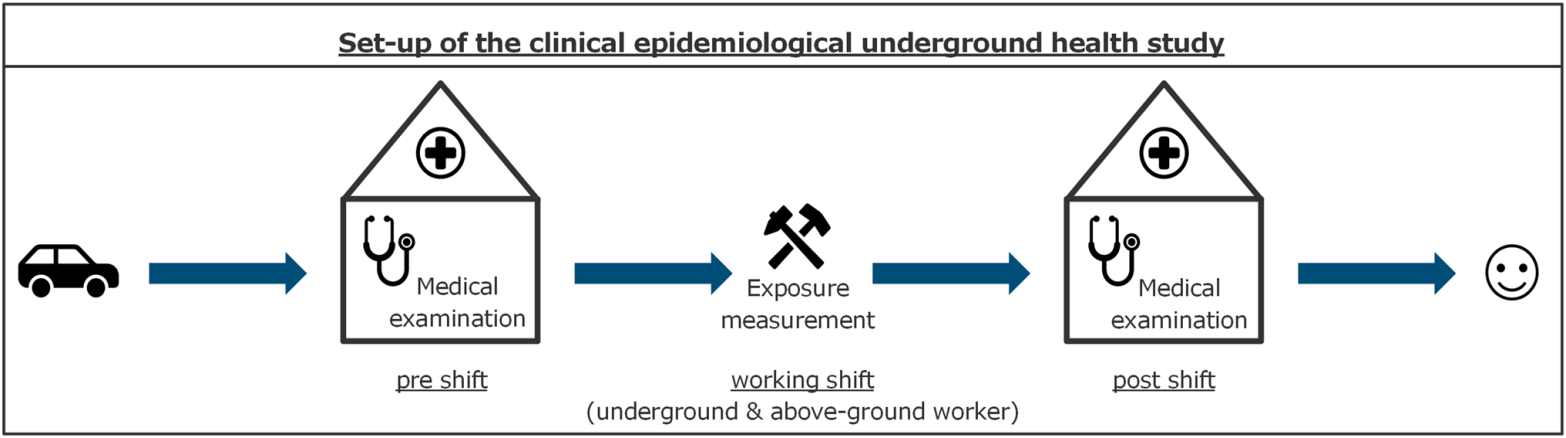
Scheme showing the study procedure each of the participants must pass through

For each participant, we based data collection on a single 8-h shift between August 2017 and January 2019. We performed medical examinations before and after the shift and collected personal samples of NO, NO_2_ and EC-DPM during the shift (Fig. 1).

Participants completed a pre-shift survey which included questions about smoking status (current daily smoker, non-smoker including occasional smoker (< 5 cigarettes per week), and former smoker). In pre- and post-shift medical examinations, each lasting 45 min, medical staff collected blood and urine samples and recorded blood pressure, age, height, and weight. Blood and urine samples were analyzed by an independent external medical laboratory. Lung function measurements were performed on-site using a ‘Vyntus’ body plethysmograph equipped with ‘SentrySuite^™^’ software (Vyaire, Höchstadt, Germany), and fractional concentration of exhaled nitric oxide (FeNO) was measured using a ‘Niox Vero’ device (Circassia, Bad Homburg, Germany). Quality control was performed by medical experts from the Institute for Prevention and Occupational Medicine of the German social accident Insurance, Institute of the Ruhr University Bochum (IPA).

Personal sampling for exposure to NO, NO_2_ and EC-DPM was conducted during participants’ 8-h shifts. Before entering their workplaces, the participants were equipped with a personal dust sampler (PGP and SIMPEDS-Cyclone type FSP2), connected to a sampling pump (GSA Messgerätebau, type GSA SG 5100ex) and a ‘Dräger X-am^®^ 5600’ (Lübeck, Germany) personal gas monitor for continuous measurements of NO and NO_2_. Devices were attached and removed above ground and worn during the entire shift. Due to ambient conditions in the mines such as high temperatures, devices were pretreated at 30 C and 45% relative humidity to reduce potential breakdowns. Sample pumps used for EC-DPM measurements were checked and monitored according to DIN EN ISO 13137:2014–03; ‘X-am^®^ 5600’ devices were calibrated regularly via ‘X-dock 6600’ using special calibration gases (NO, NO_2_, N_2_) purchased from ‘Air Products’. While NO and NO_2_ data were detected directly, EC-DPM was subsequently analyzed coulometrically. These analyses and overall quality control were performed by experts from the Institute for the Research on Hazardous Substances (IGF). 8-h average concentrations were derived for NO, NO_2_ and EC-DPM for each participant.

### Statistical methods

Statistical analyses were conducted using SAS 9.4 (RRID: SCR_008567; SAS Institute, Cary, NC). We checked the data for accuracy and completeness and excluded study participants with missing personal exposure data or biomarker measurements. Because most exposure and biomarker data were non-normally distributed, we used non-parametric analyses or transformed continuous variables when necessary.

We defined a categorical exposure variable, where above-ground facility workers, underground maintenance workers, and underground miners were classified as low (reference), medium, and high exposure groups, respectively. We compared personal 8-h average concentrations of NO, NO_2_ and EC-DPM among the exposure groups using non-parametric Wilcoxon rank sum tests.

For each exposure group and measurement time (pre-shift and post-shift), we estimated the median and interquartile range (IQR) as well as the mean and the standard deviation (SD) of the effect biomarkers, and we calculated the percentage of study participants whose biomarker measurements were within the reference range. Within each exposure group, we also estimated the median and IQR of the biomarkers’ post- versus pre-shift differences. We evaluated the statistical significance of these differences within each exposure group using non-parametric signed rank tests, and we evaluated divergence in the differences across the three groups using non-parametric Wilcoxon rank sum tests. In a sensitivity analysis, we compared FEV_1_%/FVC% pre-shift measurements among the three exposure groups stratified by duration of employment.

We created scatterplots to visualize the effects of personal exposure measurements on post- vs. pre-shift differences in the acute biomarkers and on pre-shift values of FEV_1_%/FVC%. We used linear regression analyses (with transformed variables where required to meet linear regression assumptions) to corroborate the graphical findings.

In all analyses, we replaced biomarker measurements below the detection limit with one-half of the detection limit. Because this method can lead to an underestimation of the variance, we conducted sensitivity analyses using imputation methods (Lotz et al. 2013). In these sensitivity analyses, we treated values below the detection limit as left-censored with the detection limit as the maximum possible value and analyzed the data using tobit regression assuming log-normal distributions.

## Results

The study included 1246 male employees (243 above-ground workers, 202 underground maintenance workers, and 801 miners) who had worked for at least one year at one of the two salt and potash mining sites (Table 1). Underground workers who participated in the study represented 50% of the company’s total underground employees. Above-ground workers had a slightly longer duration of employment and were slightly older, on average, than underground maintenance workers or miners (Table 1). Median body mass index values were similar among the three exposure groups, but the proportion of daily smokers was higher among miners, suggesting a potential confounding effect (Table 1).

**Table 1:** Characteristics of study participants (*n* = 1,246) by exposure group

Parameter	Above-ground facility	Underground	
	Reference	Maintenance	Mining
Number of study participants [*N*]			
Both sites	243	202	801
Site A	99	100	484
Site B	144	102	317
Age of study participants [years]			
Median (IQR)	40.0 (18.0)	40.0 (17.0)	37.0 (17.0)
Body mass index [kg/m^3^]			
Median (IQR)	28.4 (5.9)	27.2 (4.7)	27.3 (5.4)
Smoking behavior of study participants [Number (%)]			
Non-smoker	110 (45.3)	101 (50.3)	342 (43.0)
Former smoker	63 (25.9)	45 (22.4)	177 (22.2)
Current smoker	70 (28.8)	55 (27.4)	277 (34.8)
Duration of employment [years]			
Median (IQR)	14.4 (22.4)	12.7 (15.5)	11.9 (13.9)

Non-participants were similar to participants in terms of age and employment duration (Table S1 of the Supplementary Information, SI). Data for non-participants were only available in the aggregate. Therefore, they could not be divided into exposure groups.

### Exposure

As expected, above-ground workers had the lowest levels of 8-h mean NO and NO_2_, and absolute EC-DPM while the highest levels were seen among miners (Table 2). Median values of NO and NO_2_ did not exceed the respective OELs even among miners (NO: 1.16 ppm; NO_2_: 0.31 ppm; Table 2); median values for EC-DPM exposures only exceeded the OEL in the mining group (EC-DPM: 0.06 mg/m^3^).

**Table 2:** Median (IQR) values of exposure data for 8-h mean NO and NO_2_ as well as absolute EC-DPM by exposure group

Parameter	Above-ground facility	Underground	
	Reference	Maintenance	Mining
NO exposure, 8 h mean (OEL: 2.0 ppm) [ppm]			
Median (IQR)	0.00 (0.00)	0.42 (0.55)	1.16 (1.12)
NO_2_ exposure, 8 h mean (OEL: 0.5 ppm) [ppm]			
Median (IQR)	0.00 (0.00)	0.11 (0.25)	0.31 (0.50)
EC-DPM exposure, absolute (OEL: 0.05 mg/m^3^) [mg/m^3^]			
Median (IQR)	0.01 (0.01)	0.03 (0.03)	0.06 (0.06)

**Fig. 2:**
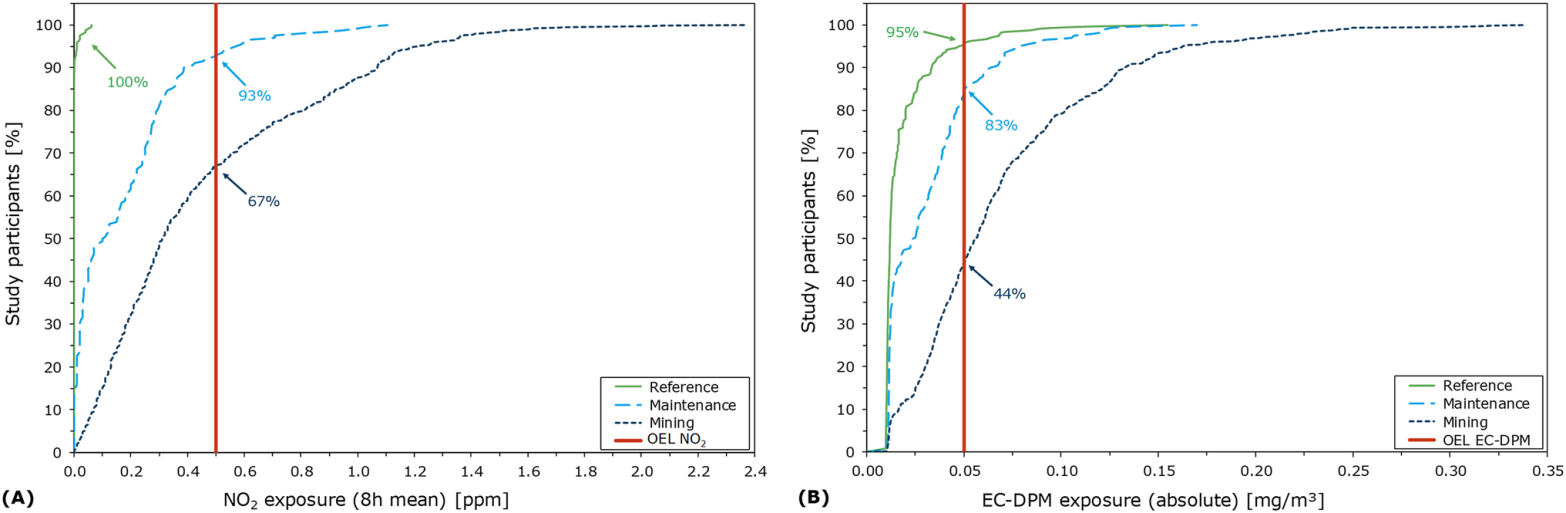
Proportion of study participants by 8-h mean exposure measurements of NO_2_ **(A)** and absolute EC-DPM exposure **(B)**, by work type

Proportions of study participants with 8-h mean NO and NO_2_, and absolute EC-DPM levels below the respective OELs were highest among above-ground workers and lowest among miners. Specifically, among above-ground workers 100% of 8-h mean NO_2_ levels (Fig. 2A) and 95% of absolute EC-DPM levels (Fig. 2B) were below the OEL. Among underground maintenance workers, 93% of 8-h mean NO_2_ levels (Fig. 2A) and 83% of absolute EC-DPM levels (Fig. 2B) were below the OEL. The proportions of underground miners with mean NO_2_ levels and absolute EC-DPM levels below the OEL were 67% and 44%, respectively (Fig. 2A, B). Results for NO were similar to those for NO_2_ and are not shown.

### Effect biomarkers across exposure groups

Pre-and post-shift measurements of the biomarkers were within the reference range for the majority of participants, regardless of exposure group (Table 3). Among cardiovascular biomarkers, pre- and post-shift values were within the reference range for > 81% of PTT measurements, for > 93% of thrombocyte measurements, and for > 72% of systolic and diastolic blood pressure (SBP and DBP) measurements. Among biomarkers of inflammation and immune response, pre- and post-shift values were within the reference range for > 88% of neutrophil, IL-6, IL-8, and TNF-α measurements, for > 80% of MPO and CRP measurements, and for > 68% of IgE measurements. No reference range was available for CC16. The proportion of FeNO measurements below the cut-off point, indicating the absence of inflammatory events in the lung, exceeded 81%. Furthermore, > 98% of pre-shift and > 99% of post-shift measurements of FEV_1_%/FVC% were within the normal physiological range.

**Table 3:** Percentage of study participants with pre- and post-shift biomarker measurements below, within and above the reference range (RR) and above or below the cut-off point, by exposure group

Group of biomarkers	Biomarkers		Above-ground facility		Underground				Reference range (RR) / cut-off point
			Reference [%]		Maintenance [%]		Mining [%]		
			Pre shift	Post shift	Pre shift	Post shift	Pre shift	Post shift	
Cardiovascular biomarkers	PTT	< RR	0.9	0.9	2.0	2.5	2.0	1.5	26–36 s
		= RR	81.4	84.8	84.2	83.3	82.5	82.7	
		> RR	17.8	14.4	13.8	14.2	15.5	15.8	
	Thrombocytes	< RR	3.4	2.9	4.2	1.5	3.1	3.1	150–361 ^1^/nl
		= RR	95.8	95.4	93.8	96.9	96.1	96.3	
		> RR	0.9	1.7	2.1	1.5	0.9	0.6	
	SBP	< RR	1.2	0.4	2.0	1.0	1.5	0.4	105–139 mmHg
		= RR	79.4	74.9	76.7	74.8	73.7	74.0	
		> RR	19.4	24.7	21.3	24.3	24.8	25.7	
	DBP	< RR	1.6	1.6	1.5	0.5	1.4	1.6	65–89 mmHg
		= RR	72.4	77.0	81.2	78.7	73.2	73.6	
		> RR	25.9	21.4	17.3	20.8	25.5	24.8	
Biomarkers of inflammation and immune response	Neutrophils	< RR	11.2	8.9	11.5	4.6	9.2	4.5	42.0–76.0%
		= RR	88.8	91.1	88.5	96.4	90.8	95.5	
	IL-6	= RR	96.6	97.9	97.5	97.5	97.7	97.5	0.0–7.0 pg/ml
		> RR	3.4	2.1	2.5	2.5	2.3	2.5	
	IL-8	= RR	100.0	100.0	100.0	100.0	100.0	100.0	0.0–62.0 pg/ml
		> RR	0.0	0.0	0.0	0.0	0.0	0.0	
	TNF-α	= RR	88.7	91.7	87.8	91.0	88.1	90.9	0.0–8.1 pg/ml
		> RR	11.3	8.3	12.2	9.05	11.9	9.1	
	MPO	= RR	90.3	90.4	88.3	80.0	86.5	83.8	0.0–400.0 ng/ml
		> RR	9.7	9.6	11.7	20.1	13.5	16.2	
	CC16	= RR	NA	NA	NA	NA	NA	NA	NA
	CRP	= RR	88.4	88.4	91.0	91.5	91.1	90.1	0.0–5.0 mg/ml
		> RR	11.6	11.6	9.1	8.5	8.9	10.0	
	IgE	= RR	72.3	72.7	70.9	70.4	68.9	70.0	0.0–100.0 IU/ml
		> RR	27.7	27.3	29.2	29.7	31.1	30.0	
Respiratory biomarkers	FeNO	< cut-off	86.8	90.5	81.6	84.5	88.1	89.2	< 25.0 ppb
		> cut-off	13.2	9.5	18.4	15.5	11.9	10.8	
	FEV_1_%/FVC%	< cut-off	1.3	0.8	0.0	0.0	0.4	0.6	> 70%
		> cut-off	98.7	99.2	100.0	100.0	99.6	99.4	

Median differences between post- and pre-shift measurements of cardiovascular biomarkers and biomarkers of inflammation and immune response tended to be small, and were zero for PTT, SBP and DBP and for IL-6, IL-8, and CRP in all three exposure groups (Table 4). Additional information on mean values is given as background information in the supporting information (Table S3). For thrombocytes, post-shift measurements exceeded pre-shift values within each exposure group (all signed rank test *p*-values < 0.0001), but the differences were similar across the exposure groups (Wilcoxon rank sum test *p*-value > 0.2).

**Table 4:** Number *N* and median (IQR), pre shift, post shift and post- vs. pre-shift differences in cardiovascular biomarkers and biomarkers of inflammation and immune response, by exposure group; and number *N* and median (IQR), pre shift, post shift and post- vs. pre-shift differences of FeNO as well as pre- and post-shift measurements of FEV_1_%/FVC%, by smoking status and exposure group

Group of biomarkers	Biomarkers	Above-ground facility				Underground							
		Reference				Maintenance				Mining			
		N^*^	Median (IQR)			N^*^	Median (IQR)			N^*^	Median (IQR)		
			Pre shift	Post shift	post- vs. pre-shift		Pre shift	Post shift	post- vs. pre-shift		Pre shift	Post shift	Post- vs. pre-shift
Cardiovascular biomarkers	PTT [s]	229	32.0 (5.0)	32.0 (5.0)	0.0 (3.0)	193	32.0 (5.5)	32.0 (5.0)	0.0 (2.0)	765	32.0 (5.0)	32.0 (5.0)	0.0 (3.0)
	Thrombocytes [^1^/_nl_]	231	229.5 (63.5)	237.5 (60.0)	7.0 (19.0)	187	234.5 (74.0)	241.0 (74.0)	6.0 (20.0)	770	235.0 (60.0)	240.0 (61.0)	4.0 (19.0)
	SBP [mmHg]	243	125.0 (10.0)	125.0 (15.0)	0.0 (15.0)	202	125.0 (10.0)	125.0 (10.0)	0.0 (15.0)	799	130.0 (15.0)	130.0 (20.0)	0.0 (20.0)
	DBP [mmHg]	243	80.0 (10.0)	80.0 (5.0)	0.0 (10.0)	202	80.0 (0.0)	80.0 (5.0)	0.0 (5.0)	799	80.0 (10.0)	80.0 (5.0)	0.0 (10.0)
Biomarkers of inflammation and immune response	Neutrophils [%]	228	52.0 (13.0)	55.0 (10.0)	2.0 (14.0)	187	51.5 (11.5)	54.0 (10.0)	4.0 (11.0)	762	53.0 (12.0)	55.0 (11.0)	2.0 (14.0)
	IL-6 [pg/ml]	236	0.8 (1.5)	1.6 (1.7)	0.0 (0.8)	196	0.8 (1.5)	0.8 (1.5)	0.0 (0.6)	785	0.8 (1.5)	1.5 (1.8)	0.0 (1.0)
	IL-8 [pg/ml]	236	6.0 (5.5)	6.0 (5.5)	0.0 (3.0)	196	6.0 (5.5)	6.0 (5.5)	0.0 (3.0)	784	6.0 (5.5)	6.0 (5.5)	0.0 (3.0)
	TNF-α [pg/ml]	236	5.5 (2.4)	5.4 (2.5)	-0.3 (1.6)	196	6.1 (2.1)	5.6 (2.1)	-0.4 (1.5)	785	6.0 (2.2)	5.7 (2.1)	− 0.3 (1.3)
	MPO [ng/ml]	234	231.6 (112.7)	259.9 (117.2)	19.2 (101.4)	196	243.3 (140.7)	278.8 (146.0)	32.9 (110.1)	785	246.9 (144.0)	260.4 (147.9)	16.4 (106.3)
	CC16 [ng/ml]	239	8.0 (18.4)	11.7 (20.0)	0.1 (16.5)	195	16.0 (48.2)	18.6 (44.5)	1.5 (25.9)	787	12.7 (35.6)	12.6 (31.3)	0.0 (18.2)
	CRP [mg/ml]	241	1.3 (2.7)	1.3 (2.7)	0.0 (0.2)	198	1.2 (2.0)	1.1 (1.8)	0.0 (0.2)	789	1.2 (1.8)	1.2 (1.9)	0.0 (0.2)
	IgE [IU/ml]	241	42.5 (95.0)	41.5 (97.0)	− 1.0 (4.0)	198	45.0 (105.0)	46.0 (102.0)	− 1.0 (3.0)	789	49.0 (110.0)	47.0 (106.0)	− 1.0 (4.0)
Respiratory biomarkers	FeNO [ppb]												
	Non- Smoker	109	14.0 (13.0)	13.0 (10.0)	− 1.0 (4.0)	99	17.0 (12.5)	14.0 (12.0)	− 1.0 (4.0)	337	15.0 (11.0)	14.0 (10.0)	− 1.0 (5.0)
	Former smoker	62	13.0 (11.0)	12.0 (11.0)	− 1.0 (4.0)	45	17.0 (14.0)	15.0 (14.0)	− 1.0 (4.0)	176	15.0 (11.0)	13.0 (10.0)	− 1.0 (4.0)
	Current smoker	70	9.5 (8.0)	8.0 (6.0)	− 1.0 (3.0)	54	10.0 (10.0)	10.0 (9.0)	0.0 (2.0)	274	9.0 (7.0)	8.0 (6.0)	− 1.0 (3.0)
	FEV_1_%/FVC% [%] (pre and post shift)												
	Non- smoker	106	96.0 (9.0)	95.0 (9.0)	–	96	97.0 (7.5)	98.0 (7.5)	–	316	96.0 (9.0)	96.0 (8.0)	–
	Former smoker	62	98.0 (10.0)	99.0 (8.0)	–	41	96.5 (10.0)	97.0 (10.0)	–	161	94.0 (8.0)	95.0 (9.0)	–
	Current smoker	68	94.0 (8.5)	94.5 (8.5)	–	46	93.5 (7.0)	95.0 (8.0)	–	239	95.0 (10.0)	94.5 (10.0)	–

^*^Data for number (N) are related to post- vs. pre-shift differences (except for FEV_1_%/FVC%). However, number (*N*) related to pre- and post-shift are comparable to those for post- vs. pre-shift differences

Similarly, for neutrophils, post-shift measurements were statistically significantly higher than pre-shift measurements in all three exposure groups based on signed rank tests (*p* = 0.04 for above-ground workers, *p *= 0.0004 for maintenance workers, and *p* = 0.0002 for miners) but the differences were similar across the exposure groups. Post-shift measurements were also higher than pre-shift measurements for MPO (signed rank test *p*-values < 0.0001 for all three exposure groups) but pre-shift levels exceeded post-shift levels for TNF-α and IgE (all signed rank test *p*-values < 0.0001).

For CC16, pre-shift levels were lower than post-shift levels among above-ground and maintenance workers but not among miners; none of the differences were statistically significant at the 0.05 level. Post- vs. pre-shift differences did not depend statistically significantly on exposure group (Wilcoxon rank sum test *p*-values > 0.05).

Results for the respiratory biomarkers, stratified by smoking, suggested that smoking status had a nominal effect (Table 4). For FeNO, pre-shift measurements exceeded post-shift measurements in all three exposure groups (signed rank test *p*-values < 0.0001) except for current smoker of the maintenance workers, but the differences did not depend on the exposure groups (Wilcoxon rank sum test *p*-value > 0.05). Pre-shift measurements of FEV_1_%/FVC% were similar among the three exposure groups; this finding did not change when the analysis was stratified by duration of employment (results not shown). Results for FEV_1_% and FVC% are presented in Table S2 of SI as background information.

### Associations between personal exposure measurements and effect biomarkers

Associations between personal measurements of each of the three exposure variables (NO, NO_2_ and EC-DPM) and all biomarkers did not indicate exposure-related health effects among underground workers. Because results for NO and NO_2_ were nearly identical, only results for NO_2_ are shown.

**Fig. 3:**
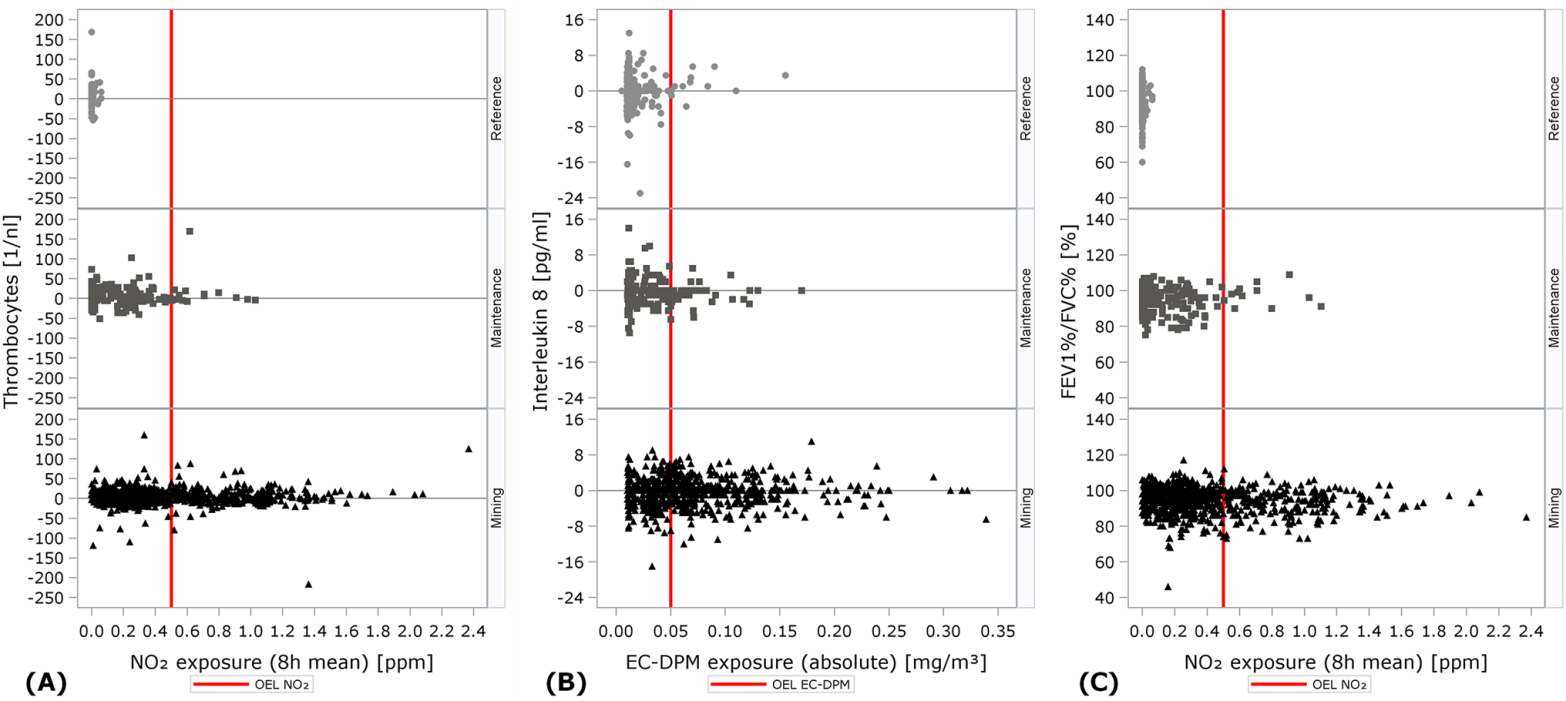
Post- vs. pre-shift differences of thrombocyte levels by 8-h mean exposure of NO_2_ **(A)**, of Interleukin 8 levels by absolute EC-DPM exposure **(B)** and pre-shift values of FEV_1_%/FVC% by 8-h mean exposure of NO_2_ **(C)** for miners, maintenance workers, and reference group workers

Scatterplots showed consistently that biomarker values (post- vs. pre-shift differences for acute biomarkers and pre-shift values for FEV_1_%/FVC%) did not depend on exposure levels, even for exposure levels exceeding the OEL. *p*-values from linear regression analyses (with variables transformed when necessary to meet linear regression assumptions) corroborated these findings. Three scatterplot exemplars are shown in Fig. 3 and in Fig. S1 of SI).

Thrombocytes were the only cardiovascular biomarker that differed post- vs. pre-shift, and differences in thrombocytes were therefore most likely to depend on exposure. However, post- vs. pre-shift differences clustered symmetrically around approximately 7 /_nl_ for all three exposure groups, with most differences ranging from about −50 /_nl_ to about + 75 /_nl_. The ranges of the differences decreased with increasing NO_2_ (Fig. 3A) and EC-DPM exposure (Fig. S1A of SI), but no consistent increase or decrease in the differences was observed even for exposure levels exceeding the OEL. All linear regression *p*-values were highly non-significant (results not shown).

IL-8 served as an example of a biomarker with a median post- vs. pre-shift difference of zero. When plotted against EC-DPM (Fig. 3B) and NO_2_ (Fig. S1B of SI), the range of post- vs. pre-shift differences was large for low exposure levels but decreased with increasing exposures. No consistent increase or decrease in the post- vs. pre-shift differences was observed in any of the three exposure groups even for exposure levels exceeding the OEL, and all linear regression *p*-values were highly non-significant (results not shown).

FEV_1_%/FVC% pre-shift values did not depend on NO_2_ (Fig. 3C) or EC-DPM (Fig. S1C of SI), even for exposure levels exceeding the OEL. Linear regression *p*-values were highly non-significant except among former smokers in the mining group (results not shown).

## Discussion

In 2016, the OELs for NO_X_ were significantly reduced to 0.5 ppm (NO_2_) and 2.0 ppm (NO) by the German regulatory authorities. For diesel particulate matter, measured as elemental carbon (EC-DPM), a new OEL was set to 50 µg/m^3^ in 2017 (Ausschuss für Gefahrstoffe 2021). The aim of the present study was to evaluate associations between workplace exposures and acute biomarkers of cardiovascular diseases, inflammation and immunological responses, and respiratory diseases among underground workers of a German salt and potash mining company. As a conservative approach, we assumed that underground workers were exposed to current levels of exhaust fumes of machines and vehicles and to blasting fumes even after initiation of a transitional action plan to reduce exposures.

We compared post- and pre-shift measurements of acute effect biomarkers among 801 miners, 202 underground maintenance workers, and 243 workers from above-ground facilities. We also investigated associations between these post- versus pre-shift differences and personal NO, NO_2_ and EC-DPM exposures measured during the shift. Most biomarker measurements were within their respective reference ranges, even among miners. While we observed statistically significant post- versus pre-shift differences for some biomarkers, the differences did not depend on the exposure group. This was the case even though about one-third of miners were exposed to NO_2_ above the current OEL in Germany (0.5 ppm) and two-thirds were exposed to EC-DPM above the OEL of 0.05 mg/m^3^ while few of the maintenance and above-ground workers exceeded these values.

In 1995 and 1997, cross-sectional studies were conducted in the same mining company (Lotz et al. 2008). In a follow-up study in 2000, three-quarters of the employees in one location and two-thirds of the employees in the other location were reexamined. Even though an overall decrease in FEV_1_ was observed in both locations, symptoms of chronic bronchitis were diagnosed among miners in one location. Several other studies have reported increased respiratory symptoms and reduced lung function among underground salt and potash miners (Lotz et al. 1998, 2006, 2008). In addition, studies of underground railway workers have shown measurable changes in a variety of biomarkers following airborne particulate matter exposure (Loxham and Nieuwenhuijsen 2019). Results from these studies may be informative but data were unavailable and direct comparisons with the findings reported here were not possible. Modest changes occurring within normal reference ranges may have little clinical short- or long-term significance. Furthermore, reliable investigations of specific associations between exposures and health effects were impossible due to strong correlations among workplace exposures (Lotz 2006). Most importantly, because underground exposures in salt and potash mines decreased by more than 50% over the last 25 years (from 0.93 ± 0.48 ppm NO_2_ and 0.15 ± 0.06 mg/m^3^ EC-DPM (Lotz et al. 2008) to 0.31 ± 0.50 ppm NO_2_ and 0.06 ± 0.06 mg/m^3^ EC-DPM (Table 2)), health effects reported in earlier studies may reflect historical exposures.

### Biomarkers of effect and exposure–response association

#### Cardiovascular biomarkers

Thrombogenicity represents a major mechanism of cardiovascular disease, both at the central (myocardial infarction) and peripheral level (e.g., stroke). In studies of air pollution, platelet counts have been used as a biomarker of thrombogenicity. Partial thromboplastin time can provide useful information on whether exposure is associated with alterations in the coagulation process (Poursafa and Kelishadi 2010). There is a known circadian influence in the coagulation system (Budkowska et al. 2019), which might be relevant for workers from the morning shift. In the present study, the majority of pre- and post-shift PTT levels were within the reference range and post- versus pre-shift differences in PTT levels were not statistically significant in any exposure group. Furthermore, there was no evidence of an association between PTT and personal exposure measurements. There was some evidence of slightly higher thrombocyte levels post-shift than pre-shift, but the increase was inversely related to personal NO_2_ and EC-DPM measurements which negated the possibility of an exposure-related inflammatory reaction. Instead, the increased post-shift values might have resulted from the physical activity of work (Dawson and Ogston 1969). We observed no change in median systolic or diastolic blood pressure after the working shift in any exposure group.

### Biomarkers of inflammation and immune response

A large number of biomarkers of inflammation and immunological response have been used in studies of air pollution, and specifically in studies of diesel exhaust. Those measured often include CRP, IL-6, IL-4, TNF-α, and MPO in serum (Hennig et al. 2014; Riedl et al. 2012; Rückerl et al. 2014). In addition, IgE levels in sputum have been measured in individuals exposed to diesel exhaust (Riedl et al. 2012). Previous studies of underground salt and potash workers also included measurements of the inflammatory serological marker CC16 (Lotz et al. 1998).

In the present study, we found no consistent evidence that the studied exposures affected CRP, IL-8, TNF-α, IgE, neutrophils, MPO, or CC16. Specifically, there was no evidence that any differences between post- and pre-shift measurements depended on exposure group or personal exposure measurements. Previous findings of increased CRP levels among miners (Sproston and Ashworth 2018) could not be confirmed.

Neutrophil count is used as a marker of thrombogenicity in clinical practice and in air pollution studies, specifically studies investigating diesel exhaust, (Epstein et al. 2013; Steenhof et al. 2014) and it can be used to assess associations between exposures and changes in the coagulation process. In the present study, we found some evidence of increased post-shift neutrophil levels in all three exposure groups. However, neutrophil levels generally were within the normal physiological range and, contrary to expectations, post- versus pre-shift differences were lower among miners than among maintenance workers but comparable among miners and above-ground workers.

Changes in MPO could indicate inflammatory reactions (Schulz et al. 2018). However, we found no evidence of an association between post- versus pre-shift differences in MPO and exposure group or personal exposure measurements. This finding was expected because MPO is mostly expressed in neutrophils, which did not depend on exposure.

### Respiratory biomarkers

FeNO, a marker of respiratory inflammation or disease (Dweik et al. 2011), has been increasingly used in air pollution studies (Berhane et al. 2014). Contrary to expectations, we observed lower levels of exhaled FeNO in post- than in pre-shift measurements across all exposure groups. No associations between post- versus pre-shift differences in FeNO and exposure group or personal exposure measurements were evident. Although smoking can affect lung function and therefore, FeNO levels (Torre et al. 2008), stratification by smoking did not meaningfully change the results. Except among former smokers in the maintenance group, FeNO measurements were consistently below the normal cut-off point.

Changes in lung function may depend on long-term exposure, and we used FEV_1_%/FVC% in a sensitivity analysis to explore long-term effects. We found no evidence that FEV_1_%/FVC% measurements depended on exposure group or personal exposure measurements. As expected, in all three exposure groups, FEV_1_%/FVC% values were lower among current smokers than among non-smokers; FEV_1_%/FVC% values were lower among former smokers than among non-smokers among maintenance workers and miners. However, even among current and former smokers, FEV_1_%/FVC% values were above the normal cut-off point. Stratification by duration of employment did not change these conclusions.

### Strengths and limitations

The present study has several strengths. Based on the sample size calculations in our feasibility study, the study had adequate statistical power. Further, we selected sensitive biomarkers of acute effects and measured these biomarkers pre- and post-shift for each study participant as part of a full medical examination. This allowed for the identification of factors that might have changed during the shift and the evaluation of a possible relationship between these changes and individual exposures. In measures of body plethysmography, participants served as their own within-subject control, reducing the potential for confounding due to individual characteristics. Information on potential risk factors such as cigarette smoking, and obesity was available and effect modification could be evaluated. Potential selection bias was unlikely due to the high participation rate; one site even had more than 90% participation. This site had higher exposure levels compared to the second site, but no differences in effects between the sites were detected. Potential selection bias was not apparent based on comparisons of participants and non-participants both having similar age and employment information. Finally, due to the high number of participants predetermined by the sample size calculations in the feasibility study, there was adequate statistical power.

Limitations of the present study include the single-day cross-sectional design, which restricted the study’s ability to detect changes in biomarkers due to long-term exposure and which may have led to exposure misclassification if exposures on the study day were not representative of customary exposure levels. Furthermore, it is possible that factors other than workplace exposures (e.g., diet, medications, co-existing health problems, etc.) could have influenced the biomarker levels. However, it is unlikely that factors occurring in some individuals would have concealed a true association across the study population. Further, failure to adjust for confounding is most likely to hide a true association if the confounder is positively associated with the exposure and inversely associated with the outcome or vice versa, which was unlikely to be the case here.

## Conclusion

The aim of the present study was to determine whether exposures to current levels of NO, NO_2_ and EC-DPM are associated with sensitive indicators of acute health effects. While none of the above-ground workers exceeded the OEL for NO_2_ and only 5% exceeded the OEL for EC-DPM exposure, underground workers often exceeded the OEL for NO_2_ and EC-DPM. Hence, 33% of miners and 7% underground maintenance workers exceeded the OEL for NO_2_; the OEL for EC-DPM was exceeded by 56% of miners and 17% of maintenance workers.

We investigated cardiovascular and respiratory biomarkers as well as biomarkers of inflammation and immune response. For some biomarkers (thrombocytes, neutrophils, MPO, TNF-α, IgE, and FeNO), we observed statistically significant differences between pre- and post-shift measurements. However, we did not find any consistent associations between exposure group or individually measured exposure levels and effect biomarkers, even when exposures exceeded the German OEL. Our results did not provide evidence that workers in salt and potash mining in Germany are likely to experience measurable acute health hazards at current levels of workplace exposure.

## Supplementary Information

Below is the link to the electronic supplementary material.

Supplementary file1 (PDF 354 KB)

## Data Availability

The raw data generated and analyzed for this study are not publicly available as they include confidential personal medical and corporate data. As required by contract, the final dataset is therefore held by a trustee, the German Social Accident Insurance Institution for the Raw Materials and Chemical Industry (BG RCI) (Heidelberg, Germany).
