# Supplementary material for: Underground salt and potash workers exposed to nitrogen oxides and diesel exhaust: assessment of specific effect biomarkers
Source: Int Arch Occup Environ Health. 2022 May 18;95(9):1817–28. doi: 10.1007/s00420-022-01876-2 (PMC9630174; doi:10.1007/s00420-022-01876-2)
Supplement: Supplementary file 1 — Supplementary file1 (PDF 354 KB) [file 420_2022_1876_MOESM1_ESM.pdf]

Supplementary Information (SI)

International Archives of Occupational and Environmental Health

Underground Salt and Potash Workers exposed to Nitrogen Oxides and Diesel Exhaust – Assessment of specific Effect Biomarkers

Lisa Gamrad-Streubel[1] (https://orcid.org/0000-0001-9694-5057) – Lisa-Marie Haase[1] – Katharina K. Rudolph[1] – Katrin Rühle[1] – Annette M. Bachand[2] (https://orcid.org/0000-0002-5265-1572) – Lori Crawford[2] (https://orcid.org/0000-0003-0972-4104) – Kenneth A. Mundt[3] (https://orcid.org/0000-0002-9700-8679) – Jürgen Bünger[4] (https://orcid.org/0000-0001-6831-7854) – Dirk Pallapies[4] – Dirk Taeger[4] (https://orcid.org/0000-0002-5340-5584) – Swaantje Casjens[4] (https://orcid.org/0000-0002-0148-8859) – Anja Molkenthin[4] – Savo Neumann[4] – Jörg Giesen[5] – Volker Neumann[5] – Thomas Brüning[4] – Thomas Birk[1] (https://orcid.org/0000-0003-3882-7743)

1 Ramboll Deutschland GmbH, Environment & Health, Essen, Germany
2 Ramboll US Consulting, Inc., Amherst, Massachusetts, USA
3 Cardno ChemRisk, Boston, Massachusetts, USA
4 Institute for Prevention and Occupational Medicine of the German Social Accident Insurance, Institute of the Ruhr University Bochum (IPA), Bochum, Germany
5 Institute for the Research on Hazardous Substances (IGF), Bochum, Germany

Corresponding Author

Lisa Gamrad-Streubel
Ramboll Deutschland GmbH
City Tower - Limbecker Platz 1
45127 Essen
Germany
lgamrad@ramboll.com

Table S1 Duration of employment and age of non-participants

Parameter	Duration of employment of non-participants [Years]	Age of non-participants [Years]
N	1456	1456
Median (IQR)	14.6 (24.2)	43.7 (21.4)

Table S2 Number N, median (IQR) and mean (SD) pre- and post-shift measurements of FEV_1% and FVC%, by smoking status and exposure group

Group of biomarkers	Biomarkers	Above-ground facility			Underground					
		Reference			Maintenance			Mining		
		N	Median (IQR)	Mean (SD)	N	Median (IQR)	Mean (SD)	N	Median (IQR)	Mean (SD)
Respiratory biomarkers	FEV_1% [%] (pre shift)									
	Non-smoker	106	94.0 (17.0)	94.9 (12.2)	96	99.5 (17.5)	97.2 (12.8)	316	96.0 (14.5)	95.0 (11.3)
	Former smoker	62	99.0 (18.0)	98.0 (14.4)	41	93.0 (16.0)	90.8 (11.7)	161	93.0 (15.0)	93.3 (11.0)
	Current smoker	68	91.0 (11.0)	90.8 (11.9)	46	94.0 (11.0)	93.1 (10.7)	239	92.0 (13.0)	92.3 (10.6)
	FEV_1% [%] (post shift)									
	Non-smoker	106	92.0 (19.0)	94.3 (12.1)	96	99.0 (15.5)	97.4 (13.1)	316	95.5 (15.0)	94.9 (11.7)
	Former smoker	62	98.4 (18.0)	98.1 (14.3)	41	91.0 (16.0)	90.7 (11.1)	161	94.0 (16.0)	94.1 (11.0)
	Current smoker	68	90.0 (13.0)	90.1 (11.0)	46	94.5 (12.0)	94.4 (10.0)	239	93.0 (14.0)	92.3 (10.4)
	FVC% [%] (pre shift)									
	Non-smoker	106	96.0 (17.0)	96.7 (11.9)	96	100.0 (14.0)	99.3 (10.7)	316	98.0 (14.0)	98.1 (10.3)
	Former smoker	62	98.5 (14.0)	99.9 (11.8)	41	96.0 (13.0)	94.4 (10.0)	161	98.0 (13.0)	97.5 (10.3)
	Current smoker	68	96.0 (9.5)	95.9 (9.1)	46	96.0 (17.0)	99.2 (11.4)	239	98.0 (13.0)	97.2 (9.6)
	FVC% [%] (post shift)									
	Non-smoker	106	95.5 (17.0)	96.4 (11.9)	96	100.0 (14.0)	99.0 (11.1)	316	98.0 (14.0)	97.9 (10.4)
	Former smoker	62	99.0 (14.0)	99.4 (11.4)	41	95.0 (10.0)	94.4 (10.0)	161	97.0 (15.0)	97.9 (9.9)
	Current smoker	68	95.0 (9.0)	94.9 (9.1)	46	100.5 (18.0)	99.9 (11.3)	239	97.0 (14.0)	97.0 (9.5)

Table S3 Number N and mean (SD), pre shift, post shift and post- vs. pre-shift differences in cardiovascular biomarkers and biomarkers of inflammation and immune response, by exposure group; and number N and mean (SD), pre shift, post shift and post- vs. pre-shift differences of FeNO as well as pre- and post-shift measurements of FEV_1%/FVC%, by smoking status and exposure group

Group of biomarkers	Biomarkers	Above-ground facility				Underground							
		Reference				Maintenance				Mining			
		N*	Mean (SD)			N*	Mean (SD)			N*	Mean (SD)		
			Pre shift	Post shift	post- vs. pre-shift		Pre shift	Post shift	post- vs. pre-shift		Pre shift	Post shift	post- vs. pre-shift
Cardiovascular biomarkers	**PTT [s]**	229	33.3 (5.7)	32.6 (4.2)	-0.8 (5.5)	193	32.6 (4.7)	32.8 (4.1)	0.1 (3.9)	765	32.7 (4.2)	32.6 (4.2)	-0.1 (2.9)
	**Thrombocytes [1/nl]**	231	236.2 (49.8)	242.0 (52.1)	7.0 (19.9)	187	235.8 (54.6)	244.0 (52.0)	7.3 (22.7)	770	236.7 (48.2)	241.8 (48.2)	5.2 (20.8)
	**SBP [mmHg]**	243	125.8 (12.8)	127.4 (12.1)	1.6 (12.0)	202	126.3 (12.0)	127.8 (13.0)	1.4 (12.2)	799	128.3 (12.8)	129.1 (11.6)	0.8 (13.1)
	**DBP [mmHg]**	243	81.5 (7.8)	81.2 (7.6)	-0.4 (8.5)	202	80.6 (7.3)	81.3 (7.8)	0.7 (8.3)	799	82.0 (7.8)	81.6 (7.7)	-0.3 (8.6)
Biomarkers of inflammation and immune response	**Neutrophils [%]**	228	52.9 (9.4)	53.9 (8.0)	1.0 (9.5)	187	52.1 (8.6)	54.1 (7.4)	2.0 (8.9)	762	53.5 (9.1)	54.7 (7.8)	1.1 (9.4)
	**IL-6 [pg/ml]**	236	2.1 (4.5)	2.0 (1.9)	-0.1 (4.2)	196	1.8 (1.8)	1.8 (1.6)	0.0 (1.6)	785	1.8 (2.1)	2.0 (2.3)	0.2 (1.8)
	**IL-8 [pg/ml]**	236	6.1 (3.8)	6.2 (3.6)	0.2 (3.7)	196	6.2 (2.9)	6.1 (3.2)	-0.2 (3.0)	784	6.5 (3.5)	6.1 (3.3)	-0.4 (3.0)
	**TNF-α [pg/ml]**	236	6.0 (4.7)	5.4 (3.3)	-0.7 (3.9)	196	7.3 (18.2)	5.6 (2.1)	-1.8 (18.2)	785	6.6 (17.9)	6.2 (15.3)	-0.4 (2.9)
	**MPO [ng/ml]**	234	266.6 (170.8)	297.4 (261.9)	29.3 (184.8)	196	267.0 (125.4)	307.4 (147.6)	38.4 (107.2)	785	279.4 (167.6)	300.0 (164.2)	23.8 (113.7)
	**CC16 [ng/ml]**	239	14.7 (19.3)	18.0 (29.9)	2.1 (20.0)	195	45.1 (80.1)	55.0 (105.7)	7.6 (80.9)	787	42.3 (86.9)	38.7 (80.0)	-3.5 (92.0)
	**CRP [mg/ml]**	241	2.6 (3.6)	2.5 (3.5)	-0.1 (1.2)	198	2.2 (3.2)	2.2 (3.3)	0.0 (0.8)	789	2.2 (3.7)	2.2 (3.5)	0.0 (1.1)
	**IgE [IU/ml]**	241	134.7 (319.7)	131.0 (314.1)	-4.3 (19.2)	198	101.9 (172.2)	99.9 (165.4)	-3.0 (12.0)	789	163.4 (471.8)	158.3 (465.2)	-4.2 (25.0)
Respiratory biomarkers	**FeNO [ppb]**												
	Non-smoker	109	17.7 (11.9)	16.4 (11.2)	-1.3 (4.0)	99	18.9 (10.6)	17.8 (10.9)	-1.6 (4.2)	337	17.8 (12.0)	17.1 (11.7)	-0.8 (4.7)
	Former smoker	62	17.1 (14.3)	15.2 (10.9)	-2.0 (7.1)	45	20.5 (15.7)	20.8 (18.2)	0.3 (6.1)	176	17.4 (14.5)	16.1 (13.7)	-1.2 (5.4)
	Current smoker	70	11.5 (9.3)	10.8 (9.5)	-0.8 (4.7)	54	13.2 (9.2)	11.9 (7.9)	-1.0 (3.4)	274	11.9 (10.5)	10.9 (10.3)	-1.1 (6.0)
	**FEV_1%/FVC% [%] (pre and post shift)**												
	Non-smoker	106	95.6 (7.5)	95.7 (7.1)	/	96	96.2 (7.1)	96.5 (7.3)	/	316	95.2 (7.5)	95.1 (7.9)	/
	Former smoker	62	96.6 (7.9)	96.9 (7.8)	/	41	94.8 (7.4)	94.7 (7.4)	/	161	94.0 (6.6)	94.6 (7.1)	/
	Current smoker	68	93.3 (8.8)	93.3 (7.5)	/	46	92.8 (6.3)	93.5 (6.6)	/	239	93.7 (7.5)	93.7 (7.1)	/

* Data for number (N) are related to post- vs. pre-shift differences (except for FEV_1%/FVC%). However, number (N) related to pre- and post-shift are comparable to those for post- vs. pre-shift differences.

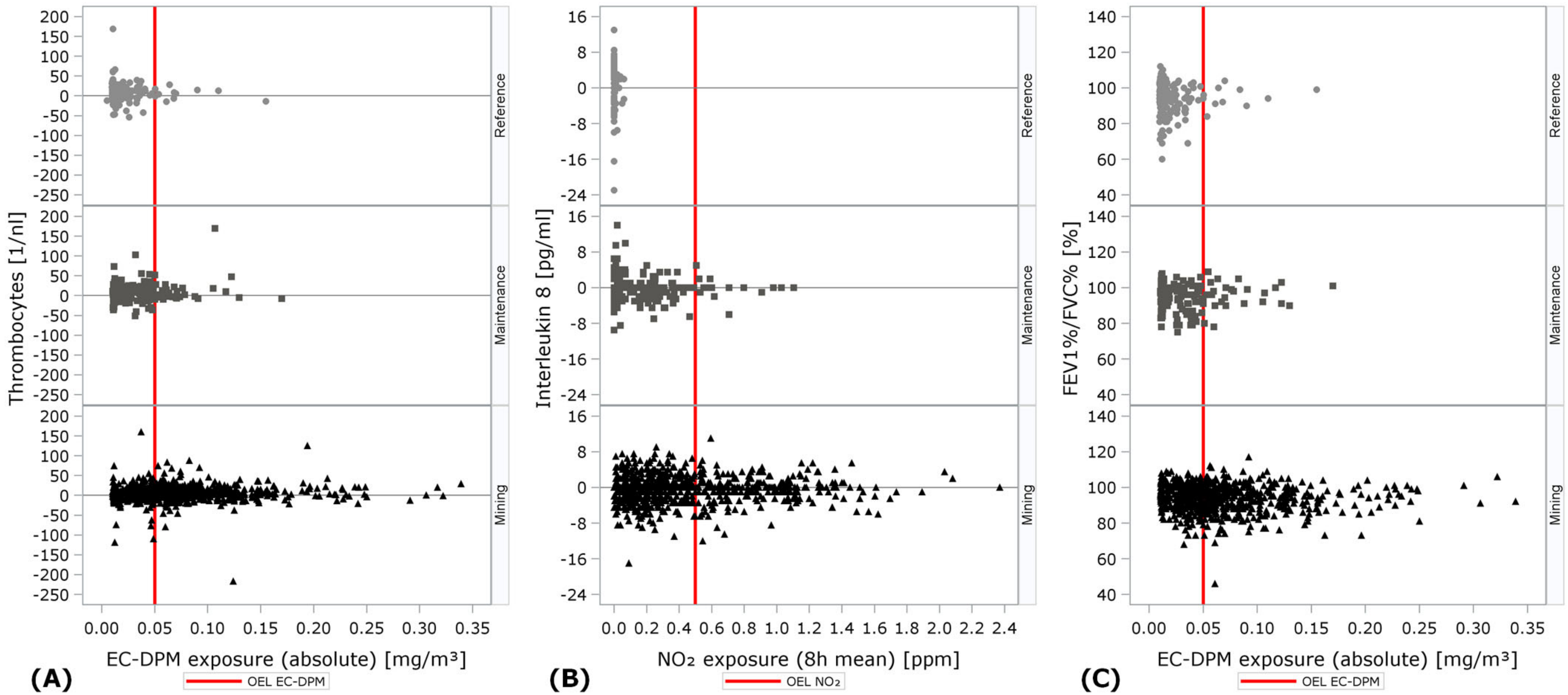

Fig. S1 Post- vs. pre-shift differences of thrombocyte levels by absolute EC-DPM exposure (A), of Interleukin 8 levels by 8-hour mean exposure of NO_2 (B) and pre-shift values of FEV_1%/FVC% by absolute EC-DPM exposure (C) for miners, maintenance workers, and reference group workers
